# Human approach-avoidance conflict behaviour relates to transdiagnostic psychiatric symptom dimensions

**DOI:** 10.1038/s41398-026-03835-8

**Published:** 2026-01-28

**Authors:** Juliana K. Sporrer, Filip Melinscak, Dominik R. Bach

**Affiliations:** 1 Max Planck UCL Centre for Computational Psychiatry and Ageing Research and Wellcome Centre for Human Neuroimaging, Queen Square Institute of Neurology, University College London, London, United Kingdom; 2 Department of Cognition, Emotion, and Methods in Psychology, Faculty of Psychology, University of Vienna, Vienna, Austria; 3 Department of Psychiatry, Psychotherapy and Psychosomatics, Psychiatry Hospital, University of Zurich, Zurich, Switzerland; 4 University of Bonn, Transdisciplinary Research Area “Life and Health”, Centre for Artificial Intelligence and Neuroscience, Bonn, Germany

**Keywords:** Learning and memory, Psychiatric disorders

## Abstract

Approach-avoidance conflict (AAC), a laboratory representation of risky foraging, serves as mainstay of pre-clinical anxiety disorder research, motivated by an impact of anxiolytic drugs on cautious behaviour. While cautiousness appears to be a stable behavioural trait, growing evidence suggests that it is not strongly related to self-reported anxiety. Here, we ask more broadly which psychiatric symptom dimensions relate to AAC behaviour, using a cross-sectional, data-driven, exploration-confirmation approach across two large online samples (N1 = 315; N2 = 690). In a previously validated task, participants chose whether, and when, to approach rewards under varying threat probability and magnitude. They then completed a comprehensive psychiatric questionnaire battery with a known three-factor structure. A broad psychopathology factor, mainly related to impulsivity and OCD symptoms and not specifically linked with anxiety, showed the strongest relation to all behavioural readouts. Higher symptom scores related to decreased passive avoidance, increased behavioural inhibition, and reduced sensitivity to threat features. This factor was also associated with an altered subjective model of threat and reward relations in the environment. Broad and unspecific associations with same directional patterns but smaller magnitudes were found between individual questionnaire scores and behaviour, underscoring the status of transdiagnostic dimensions. Crucially, no associations were found between behaviour and transdiagnostic anxiety-depression, or with gender. This study highlights that cautiousness in AAC tasks is comprised of two components, which are independently associated with transdiagnostic psychopathology but not specifically or particularly strongly with self-reported trait anxiety. Our cross-sectional findings underline the complex interplay of behavioural predispositions and psychopathology.

## Introduction

Avoiding threats to one’s integrity is a common and adaptive behaviour [1]. Yet, real-life situations often mandate pursuing rewards in circumstances that entail danger, exemplified by risky foraging. Such situations give rise to conflicting motivations to approach the reward, and avoid threat [2]. Solving this dilemma requires a careful assessment of available options, and the probabilities and magnitudes of the outcomes associated with each of them [3]. Approach-avoidance conflict (AAC) tests encapsulate this situation in a well-defined laboratory setting [4, 5]. Anxiolytic substances – i.e., drugs that reduce subjective feelings of anxiety in clinical conditions – crucially alter animals’ cautiousness in such tasks (see [6] for a review). This has led to a long history of employing them as a primary preclinical model in anxiety disorder research, and for the development of anxiolytic drugs. More recently, various different AAC tasks have been translated to humans, and validated by cross-species similarity of underlying neural substrates including their sensitivity to benzodiazepines and other anxiolytics, as well as to hippocampus lesions (see [2] for a review).

The reduction of cautious behaviour by anxiolytics has motivated an assumption that such cautious behaviour might relate to self-reported anxiety [7], and/or could potentially be used as diagnostic tests for anxiety disorders (see [2, 8] for a review). While this appears plausible, evidence that components of AAC behaviour relate to trait anxiety in the general population has turned out to be somewhat inconsistent (see [9] for a review). Specific relations of task readouts with trait anxiety reported in individual studies [10–13] were largely not replicated [11, 14–18], including in a large sample [19]. Similarly, a large-scale clinical study found no relation of AAC behaviour with anxiety disorder, depression, or substance use disorder [20]. Within the latter study [20] and replicated in independent samples [21, 22], a model parameter inferred from AAC behaviour correlated with anxiety impairment scores across this broad healthy and clinical population. However, there was no evidence for a relation of this parameter with anxiety symptoms within or between the groups of healthy people and anxiety disorder patients [20, 21]. Hence, while the different AAC tasks used in these studies may engage distinct components of cautious behaviour, explaining some heterogeneity of results, there is no robust evidence linking behavioural cautiousness with concurrent self-reported anxiety in the general population or in anxiety disorders, whether clinical or subclinical.

At the same time, at least some aspects of behavioural cautiousness appear to be relatively stable over two years in adolescents and young adults [19] and over one year in a mixed clinical sample [23]. To the extent that it is a somewhat stable trait, behavioural cautiousness is likely to predispose individuals to specific learning experiences over extended periods, compared to less cautious individuals. It is plausible to hypothesize that if such learning contributes to clinical conditions, these effects may not be limited to anxiety disorders alone. Indeed, evidence suggests that human temperament [24] and psychopathology [25] result from a complex interplay of predispositions, learning experiences, and learning styles that unfold throughout development. To contribute to unpacking this complex relationship, a cross-sectional approach can clarify the extent to which behavioural cautiousness, as a potentially stable behavioural predisposition, is concurrently related to dimensional psychopathology.

This is the question we sought to address here in in a data-driven approach and with a comprehensive psychiatric symptom battery. We capitalised on a previously validated human AAC task [12] with a simple and abstract visual design that could be presented online. In this task, a participant can decide whether, and how rapidly, to approach a reward, under risk of being virtually attacked by a predator and incurring a variable loss. As with many AAC tasks, its core indices of behavioural cautiousness are passive avoidance, i.e., rate of avoidance decisions, and behavioural inhibition, i.e., latency to initiate approach. Previous work has consistently demonstrated that in healthy individuals, both of these indices increase with growing threat probability and threat magnitude [12, 26–29]. Notably, behavioural inhibition in this task is not reward-maximising but might be optimal if the agent has assumptions about the temporal coupling of reward and threat [12]. Thus, in a secondary task, we assessed people’s implicit beliefs about such reward correlations [28]. We then asked whether passive avoidance and behavioural inhibition, and their relation to threat probability and magnitude, are linked to psychiatric symptom dimensions. For comparability with previous work with other behavioural tasks, we utilised a comprehensive clinical questionnaire battery with a known three-factor structure [30–32], previously labelled ‘Compulsive Behaviour and Intrusive Thought (CIT)’, ‘Anxious-Depression (AD)’ and ‘Social Withdrawal (SW)’. We complemented this battery with an IQ test, further anxiety questionnaires, and questionnaires found relevant in previous work on AAC tasks. As we had no prior hypothesis on which variables would relate to cautiousness, we opted for a rigorous exploration-confirmation approach. Hypotheses were first generated based on analysis of a discovery sample (N = 315), and then confirmed after pre-registration in a second sample (N = 690).

## Methods and materials

### Participants

Participants ( > 18 years old) were recruited online using Amazon Mechanical Turk (MTurk, https://www.mturk.com/). We included 315 participants in the discovery sample (149 females, mean age ± SD: 36.40 ± 11.01; October-November 2021) and 690 in the confirmation sample (338 females, mean age ± SD: 33.41 ± 9.89; February-March 2023; see Table S1). The combined sample comprised 1005 participants (487 females, mean age ± SD: 34.35 ± 9.89). Sample size was higher than required by a power analysis, in order to provide accurate estimators of effect sizes, in line with standards in the field of online research.

Participants who completed the task per protocol were included if they did not meet any of the following exclusion criteria: (1) Pressed (or did not press) the same button on the keyboard in more than 95% of the trials, implying that they did not follow task instructions, (2) Responded incorrectly to all three attention checks in the questionnaires (see SI), (3) Returned to the safe place in fewer than 50% of the trials, indicating lack of understanding of the task, (4) Performance at chance level. We excluded 189 (37%) participants out of 504 in the discovery sample and 478 (41%) participants out of 1168 in the confirmation sample (see Figure S1). Our exclusion rate, while somewhat higher than the typical 3–37% range found in a meta-analysis, is not unusual for online studies [33]. Although there are plausible concerns about the quality of behavioural data acquired online [34–37], extensive supporting analyses confirmed that our findings are unlikely to be explained by low data quality (see SI) [37].

After reading an information sheet about the experiment, participants gave their informed consent online. All procedures were in accordance with the Declaration of Helsinki and local regulations. The study was approved by the Governmental Ethics Committee (Kantonale Ethikkommission Zurich, BASEC 2016-00068). Participants were paid a base amount of $10.00 plus a bonus ranging from $2.00 to $14.50 (with a mean of $12.00) conditional on task performance and on attention checks.

### Experimental design

#### Tasks

**Fig. 1:**
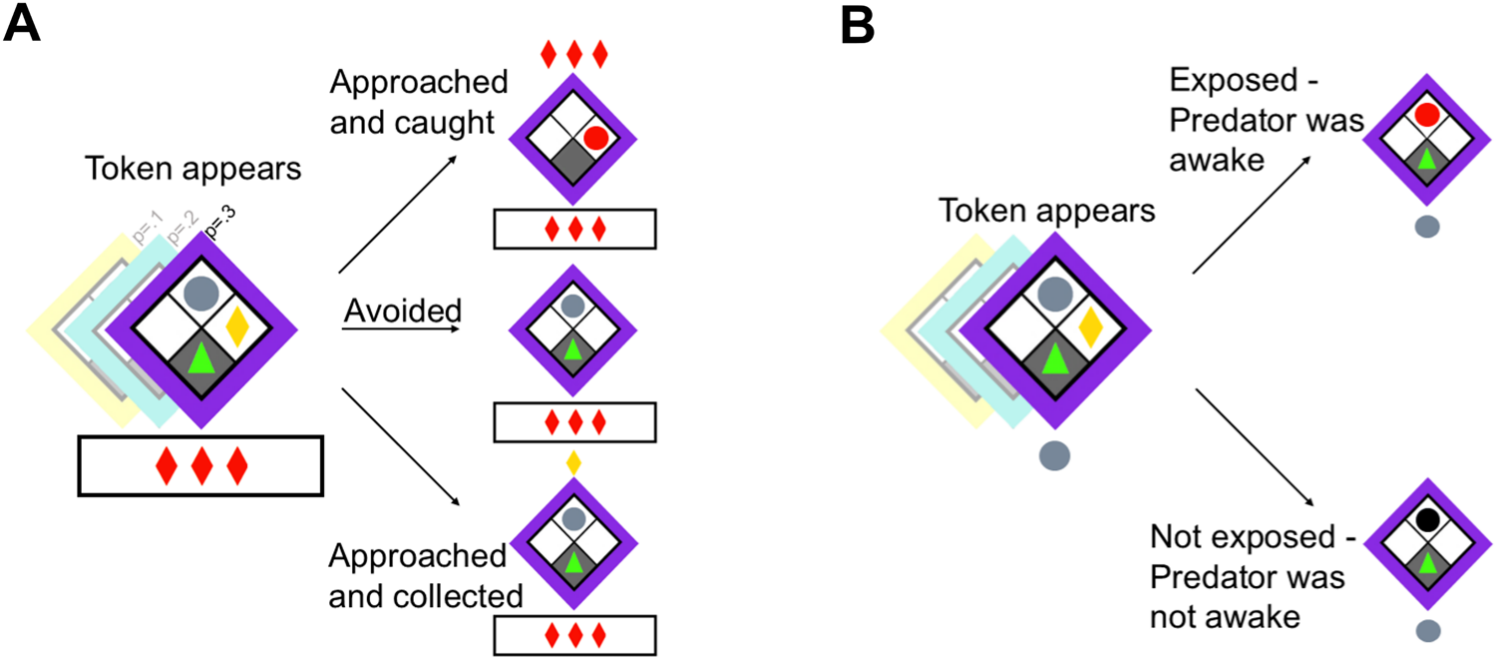
Experimental set-up for the approach-avoidance conflict task. (**A**) where participants could approach a reward at the risk of getting caught by a predator with varying levels of threat probability (shown by colour of the grid, learned by direct experience) and magnitude (signalled by the number of red tokens). In the predator exposure task (**B**), participants were asked to guess whether the predator was awake. The probability of being awake was objectively independent of time or token appearance and was randomly determined at each capture attempt.

Participants performed an AAC task (see Fig. 1) based on previous studies in a lab setting [12, 26–29, 38], see SI for details. In brief, participants could collect one monetary token (approach motivation) under threat of getting caught by a predator and consequently losing an explicitly signalled number of tokens (avoidance motivation). At the beginning of each trial, the participant started in a “safe place” at the bottom of the grid and had to decide whether to collect a token that would appear on either side after an interval. Tokens appeared after a random interval. In case the participant did not collect the token, it disappeared after a random interval. Below the grid, the potential loss of the current trial (0–5 tokens) was indicated by red tokens. A “sleeping” predator was waiting opposite the safe place in the top grid block and could catch the participant if they were outside the safe place. Three threat probabilities, corresponding to different wake-up rates (see SI), were represented by different frame colours. Crucially, threat probabilities were not explicitly instructed but it was emphasised that the predators were differently dangerous and that the participants needed to learn this difference. If the participant got caught, then the token disappeared, the predator turned red, and the indicated potential loss was realised.

This was followed by a predator exposure task, randomly interspersed with AAC task refresher trials. In this task, participants could not move outside their grid block and were instructed to expose the predator by pressing the up-arrow key (i.e. a motor action unavailable on AAC trials). If the predator was awake during the attempt, it turned red and the trial would end early. Otherwise, it would turn black and the trial would continue to the pre-determined end. This feedback allowed participants to update their knowledge of the experimental statistics (which were maintained throughout all blocks), according to which the probability of being awake was independent of time or token appearance and was randomly determined at each capture attempt. Participants were explicitly informed that the tokens were irrelevant to the task and could not be collected. If participants correctly believed that wake-up probabilities were constant, approach time would not depend on token appearance and the optimal strategy would be to approach the predator as soon as the trial starts, such as to minimize the time spent with the task. On the other hand, if participants incorrectly assumed a temporal threat-reward correlation, as suggested in previous work [12, 28], then the reward-maximizing strategy would be to approach whenever a token appeared, at the maximum of their subjective threat wake-up function.

After the two tasks, participants were asked to estimate the probability of getting caught if they left the safe place for each of the three threat levels on a continuous scale ranging from 0 to 100%. The objective threat probability for each threat level depended on behaviour and had to be implicitly learned during the experiment.

#### Psychiatric symptom questionnaires

**Fig. 2:**
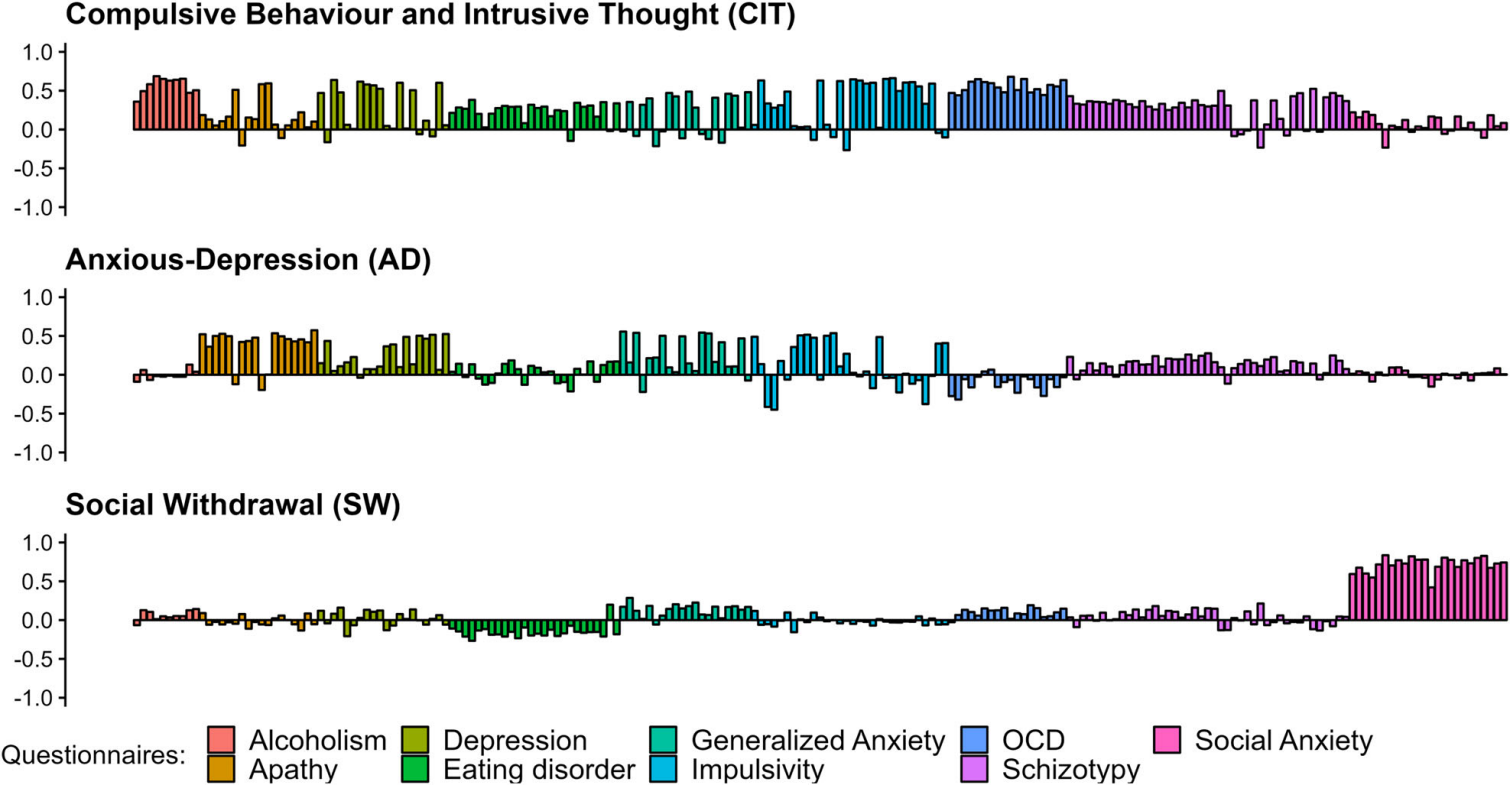
Three latent transdiagnostic symptom dimensions explained the shared variance between all questionnaire items. Loadings of all individual questionnaire items (color-coded by questionnaire) onto each factor. This plot is based on the combined sample, Figure S5 shows the results separately for the discovery and confirmation samples. See Figure S3 for the eigenvalues and correlation matrices between questionnaire in both samples.

After completion of the behavioural tasks, participants were asked to answer a battery of self-report questionnaires based on previous work [30, 31] assessing a range of psychiatric symptoms including depression (Zung Self-Rating Depression Scale) [39], generalised anxiety (Generalized Anxiety Disorder 7-item scale) [40], schizotypy (Short Scales for Measuring Schizotypy) [41], impulsivity (Barratt Impulsiveness Scale-11; [42], Obsessive-Compulsive Disorder (Obsessive-Compulsive Inventory-Revised; [43], social anxiety (Liebowitz Social Anxiety Scale) [44], eating disorders (Eating Attitudes Test) [45], apathy (Apathy Evaluation Scale) [46], alcoholism (Alcohol Use Disorders Identification Test) [47], and a short IQ evaluation (International Cognitive Ability Resource) [48]. We complemented this battery with questionnaires assessing trait anxiety (State-Trait Inventory for Cognitive and Somatic Anxiety) [49], sensation seeking (Brief Sensation Seeking Scale) [50], and the daringness subscale of the Child and Adolescent Disposition Scale [51], in order to replicate and extend previous findings with this and other AAC tasks [12, 19]. See SI for questionnaire scores (Table S1), distributions (Figure S2), and correlations (Figure S3); see Fig. 2 and Analysis Plan below for a factor analysis of the questionnaire scores.

### Analysis plan

Exclusion criteria, pre-processing steps (see SI) and analysis plan were based on a discovery sample and pre-registered before the confirmation sample was recruited (https://osf.io/5hmgk ; registered on 05 January 2023). The analysis plan encompassed 3 steps. Step 1 consisted of replicating the four behavioural group-level effects of the task previously shown in lab settings [12, 26–29]. Step 2 consisted of replicating a 3-factor solution of the transdiagnostic symptom dimensions [30–32]. For steps 1-2, each precondition needed to be confirmed at p < 0.05 to progress to the next step; hence there was no multiple comparison correction. The goal of step 3 was to assess our main research questions and investigate associations between behavioural variables of interest (indexing passive avoidance and behavioural inhibition), and psychiatric symptom dimensions extracted in step 2 and individual psychiatric questionnaires. Holm-Bonferroni method was applied to correct for multiple comparisons across 20 pre-registered hypotheses in the confirmation sample.

#### Step 1 – Replication of behavioural inhibition and passive avoidance

**Fig. 3:**
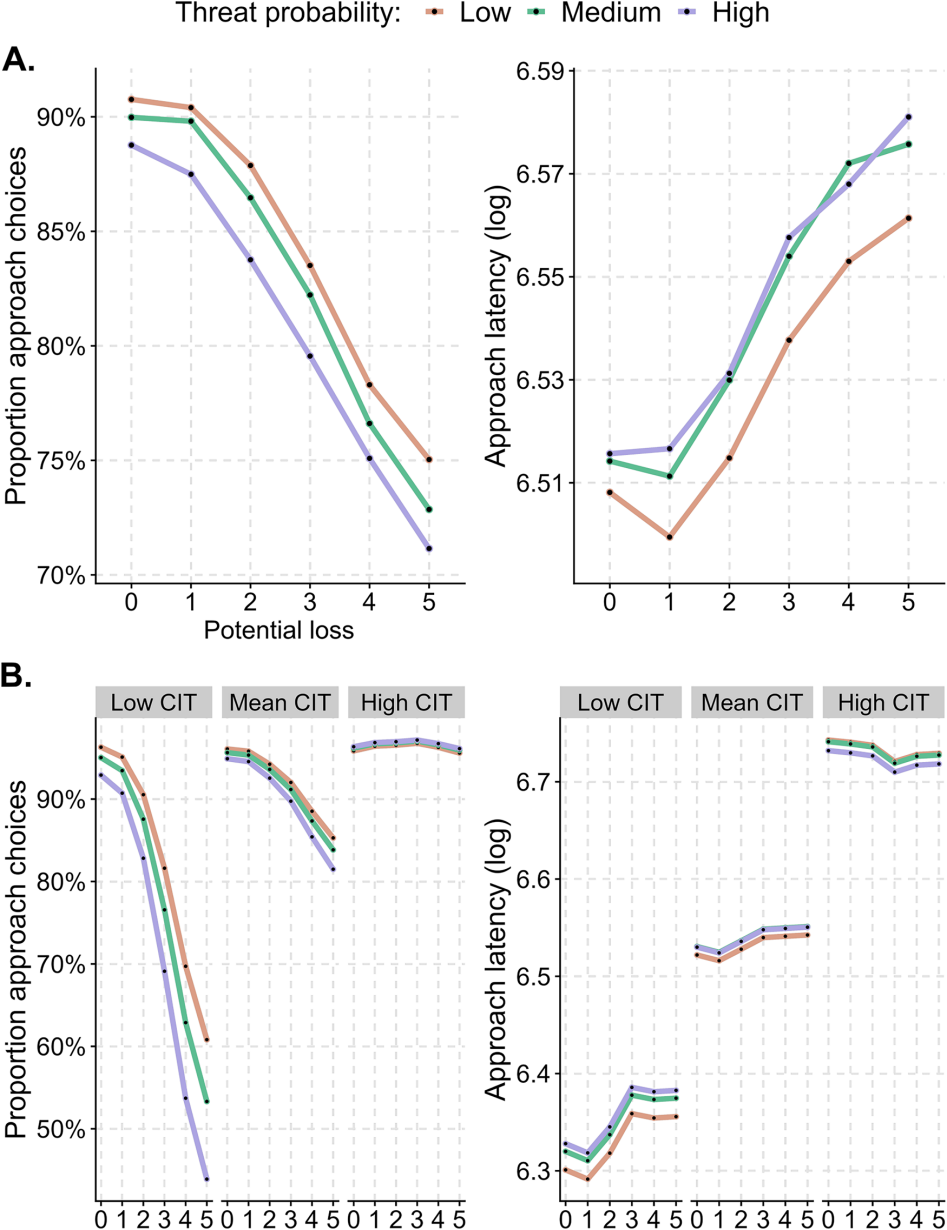
Increasing threat probability or potential loss enhance passive avoidance and behavioural inhibition. **A** Proportion of approach-avoidance decisions, indexing passive avoidance (left) and approach latency, indexing behavioural inhibition (right). **B** Estimated marginal means of approach choice (left) and latency (right) depending on CIT (Compulsive Behaviour and Intrusive Thought) symptom dimension scores while other predictors are kept fixed. This plot is based on the combined sample, Figure S7 and S8 shows the results separately for the discovery and confirmation samples. Low CIT: −1.5, Mean CIT: 0, and High CIT: +1.5.

To test whether any of the behavioural variables were influenced by the independent variables, we conducted (Generalized) Linear Mixed-Effects Models. The specification was: *Dependent variable* ~ 1 + threat level * potential loss + *(1 | participant)*. Thus, we confirmed that in line with previous lab-based results [12, 26–29, 38], passive avoidance (i.e., proportion of avoidance decisions) and behavioural inhibition (i.e. approach latency) increased with threat probability and magnitude (see Fig. 3A. and S7, Table S2).

#### Step 2 – Replication of the 3-factor psychiatric symptom dimensions

To replicate a previously established latent transdiagnostic structure [30–32], we applied an factor analysis with Maximum Likelihood Estimation and an oblique rotation on the questionnaire items (see SI for details). Exploratory (rather than confirmatory) factor analysis was chosen a priori, as we assumed that, due to differences in the sampled population, the true latent structure might be similar to—but not identical with—that found in previous studies. This revealed a 3-factor latent structure which was concordant with previous studies [30–32]. As the loadings across items had large positive correlations with the previous studies (see Figure S4), we adopted the same labels. For Factor 1 ‘Compulsive Behaviour and Intrusive Thought (CIT)’, the highest loadings came from the Alcoholism, OCD, Eating Disorders, Impulsivity, and Schizotypy questionnaires. Factor 2 ‘Anxious-Depression (AD)’ was dominated by items from the Generalised Anxiety, Depression, and Apathy questionnaires. Lastly, Factor 3 ‘Social Withdrawal (SW)’ had the highest average loadings from the Social Anxiety questionnaire, with some significant contributions from Generalised Anxiety and Eating Disorder questionnaires (see Fig. 2, S3, and S5). Exploratory inclusion of the three additional questionnaires assessing daringness, sensation seeking, and trait anxiety did not alter the 3-factor latent structure (see SI and Figure S6).

#### Step 3 – Relation of the psychiatric symptom dimensions and AAC behaviour

To test the extent to which symptom dimensions relate to AAC behaviour, we included each symptom dimension score as a z-scored fixed effect predictor into a simplified model from step 1. The specification was: *Dependent variable* ~ *1* + (threat level + potential loss) * IV + *(1 | participant)*. We also assessed the impact of age, IQ and gender, as important control variables. For all confirmed hypotheses relating to symptom dimensions, we ensured that the tests in the discovery and in the confirmation sample remained significant in a model in which age, gender and IQ were simultaneously added as covariates.

To test the extent to which the symptom dimensions relate to recollection of threat memory (i.e. learned association between colour and threat level), we included each score as a z-scored fixed effect predictor of estimated catch rates while accounting for true catch rates. The specification was:

$${Estimated}\,{catch}\,{rates} \sim 1+{actual}\,{catch}\,{rate}* {IV}+(1{|participant}).$$

Estimating explained variance in symptom dimensions is complicated by the fact that mixed-effects models include within-subjects effects and trial-by-trial data. Thus, we estimated the proportion of explained variance in a regression model with symptom dimension as dependent variable and six per-participant predictor variables: each participant’s average approach rate and latency, and their respective linear relation to threat probability and magnitude.

To investigate a subjective prior assumption that the presence of tokens alerts the predator, we estimated the influence of each symptom dimension on the percentage of predator exposure attempts before token appearance in a linear regression.

## Results

We investigated the relation between passive avoidance and behavioural inhibition on the one hand, and three transdiagnostic symptom dimensions on the other. Twenty hypotheses (denoted as H) were first generated based on a large discovery sample (N_1_ = 315) and then confirmed after pre-registration in a second sample (N_2_ = 690) with correction for multiple comparisons. To obtain the most precise population estimates, we additionally report effect sizes and inference statistics from post hoc exploratory analyses conducted on the combined sample.

### Approach-avoidance conflict decisions

We found a strong and robust relation between AAC behaviour and CIT (see Fig. 3B. and S8; Table 1). Individuals with high CIT exhibited a greater inclination to approach tokens (i.e. reduced passive avoidance, H1; p < 0.0001), such that one standard deviation increase was associated with an 59.2% increase in approach rate. Secondly, individuals with high CIT scores were delayed in approaching tokens (i.e. higher behavioural inhibition, H8; p < 0.0001), such that one standard deviation increase corresponded to a 128 ms increase in approach latency. Finally, people with high CIT had a higher tendency to make motor errors, i.e., approach the grid block opposite to the token (H20; p < 0.0001). Post-hoc analysis also revealed that individuals with high CIT were caught more often (β = 3.2, t(1, 997) = 6.71, p < 0.0001).

**Table 1:** Pre-registered hypotheses and the results of the confirmation analysis.

Hypotheses	Discovery Sample	Confirmation Sample
H1. People with high CIT approach more often	β = 0.693 (0.09), t(1, 44823) = 58.92, p < 0.0001	β = 0.367 (0.07), t(1, 98245) = 31.2, p < 0.0001
H2. The decrease in approach with higher threat level is less pronounced in people with high CIT	β = 0.267 (0.03), t(1, 44823) = 102.23, p < 0.0001	β = 0.132 (0.02), t(1, 98245) = 62.59, p < 0.0001
H3. The decrease in approach with higher potential loss is less pronounced in people with high CIT	β = 0.955 (0.04), t(1, 44823) = 546.94, p < 0.0001	β = 0.528 (0.02), t(1, 98245) = 463.76, p < 0.0001
H4. The decrease in approach with higher potential loss is more pronounced in people with high AD	β = −0.111 (0.04), t(1, 44823) = 9.64, p < 0.01	Not confirmed
H5. The decrease in approach with higher threat level is more pronounced in people with high IQ	β = −0.247 (0.02), t(1, 44966) = 112.33, p < 0.0001	β = −0.081 (0.02), t(1, 98819) = 21.21, p < 0.0001
H6. The decrease in approach with higher potential loss is more pronounced in people with high IQ	β = −0.638 (0.04), t(1, 44966) = 278.66, p < 0.0001	β = −0.182 (0.03), t(1, 98819) = 50.97, p < 0.0001
H7. The decrease in approach with higher threat level is less pronounced in males	β = 0.2 (0.05), t(1, 44680) = 17.19, p < 0.0001	Not confirmed
H8. People with high CIT approach later	β = 0.087 (0.01), t(1, 32215) = 35.54, p < 0.0001	β = 0.098 (0.01), t(1, 79978) = 114.76, p < 0.0001
H9. The increase in approach latency with higher threat level is less pronounced in people with high CIT	β = −0.014 (0.00), t(1, 32215) = 26.18, p < 0.0001	β = −0.005 (0.00), t(1, 79978) = 10.13, p < 0.01
H10. The increase in approach latency with higher potential loss is less pronounced in people with high CIT	β = −0.036 (0.00), t(1, 32215) = 72.38, p < 0.0001	β = −0.013 (0.00), t(1, 79978) = 36.09, p < 0.0001
H11. People with high AD approach earlier	β = −0.053 (0.02), t(1, 32215) = 11.97, p < 0.001	Not confirmed
H12. The increase in approach latency with higher potential loss is more pronounced in people with high AD	β = 0.012 (0.00), t(1, 32215) = 7.26, p < 0.01	Not confirmed
H13. People with high IQ approach earlier	β = −0.065 (0.02), t(1, 32335) = 18.73, p < 0.0001	Not confirmed
H14. The increase in approach latency with higher threat level is more pronounced in people with high IQ	β = 0.015 (0.00), t(1, 32335) = 25.24, p < 0.0001	Not confirmed
H15. The increase in approach latency with higher potential loss is more pronounced in people with high IQ	β = 0.023 (0.00), t(1, 32335) = 25.35, p < 0.0001	β = 0.008 (0.00), t(1, 80372) = 16.96, p < 0.0001
H16. People with high CIT overestimate catch rates more	β = 19.36 (1.49), t(1, 625) = 168.33, p < 0.0001	β = 11.11 (0.9), t(1, 1367) = 150.76, p < 0.0001
H17. For people with high CIT, estimated catch rates depend less on actual catch rates	β = −0.29 (0.04), t(1, 625) = 61.9, p < 0.0001	β = −0.15 (0.02), t(1, 1367) = 51.43, p < 0.0001
H18. People with high CIT make more predator exposure attempts before token appearance	β = 5.66, t(1, 309) = 3.19, p < 0.01	β = 6.31, t(1, 645) = 4.9, p < 0.0001
H19. People with high AD make fewer predator exposure attempts before token appearance	β = −4.88, t(1, 309) = −2.73, p < 0.01	Not confirmed
H20. People with high CIT more often approach in the incorrect direction	β = −0.426 (0.07), t(1, 33892) = 32.5, p < 0.0001	β = −0.231 (0.05), t(1, 84335) = 25.09, p < 0.0001

P-values from the discovery sample are not corrected for multiple comparison and presented as a heuristic guide only. For the confirmation sample, uncorrected P-values are presented; all of these were significant after comparing to adjusted alpha-rates according to the Holm-Bonferroni method. All confirmed hypotheses remained significant in a covariate model simultaneously controlling for age, gender, and IQ.

Next, individuals with high CIT were less sensitive to parametric threat features. The decrease in approach rate towards the token with higher threat probability (H2; p < 0.0001) and magnitude (H3; p < 0.0001) was less pronounced in people with high CIT. Similarly, the increase in approach latency with higher threat probability (H9; p < 0.01) and magnitude (H10; p < 0.0001) was less pronounced in people with high CIT. All in all, behaviour explained 37.4% variance in CIT.

The next strongest association was with IQ, with behaviour explaining 8.0% of IQ variance (see Figure S8). The decrease in approaching tokens under higher threat probability (H5; p < 0.0001) and magnitude (H6; p < 0.0001) was more pronounced in people with higher IQ. Similarly, the increase in approach latency with higher threat magnitude was more pronounced in people with high IQ (H15; p < 0.0001).

Crucially, no strong relation between behaviour and AD or gender was found, and those that were initially hypothesised were rejected in the confirmation sample (see Table 1). Indeed, no relation of any behavioural index with AD exceeded R² = 0.01 in the combined sample. Thus, in line with previous work, we found no evidence that AAC decisions related to transdiagnostic anxiety.

**Fig. 4:**
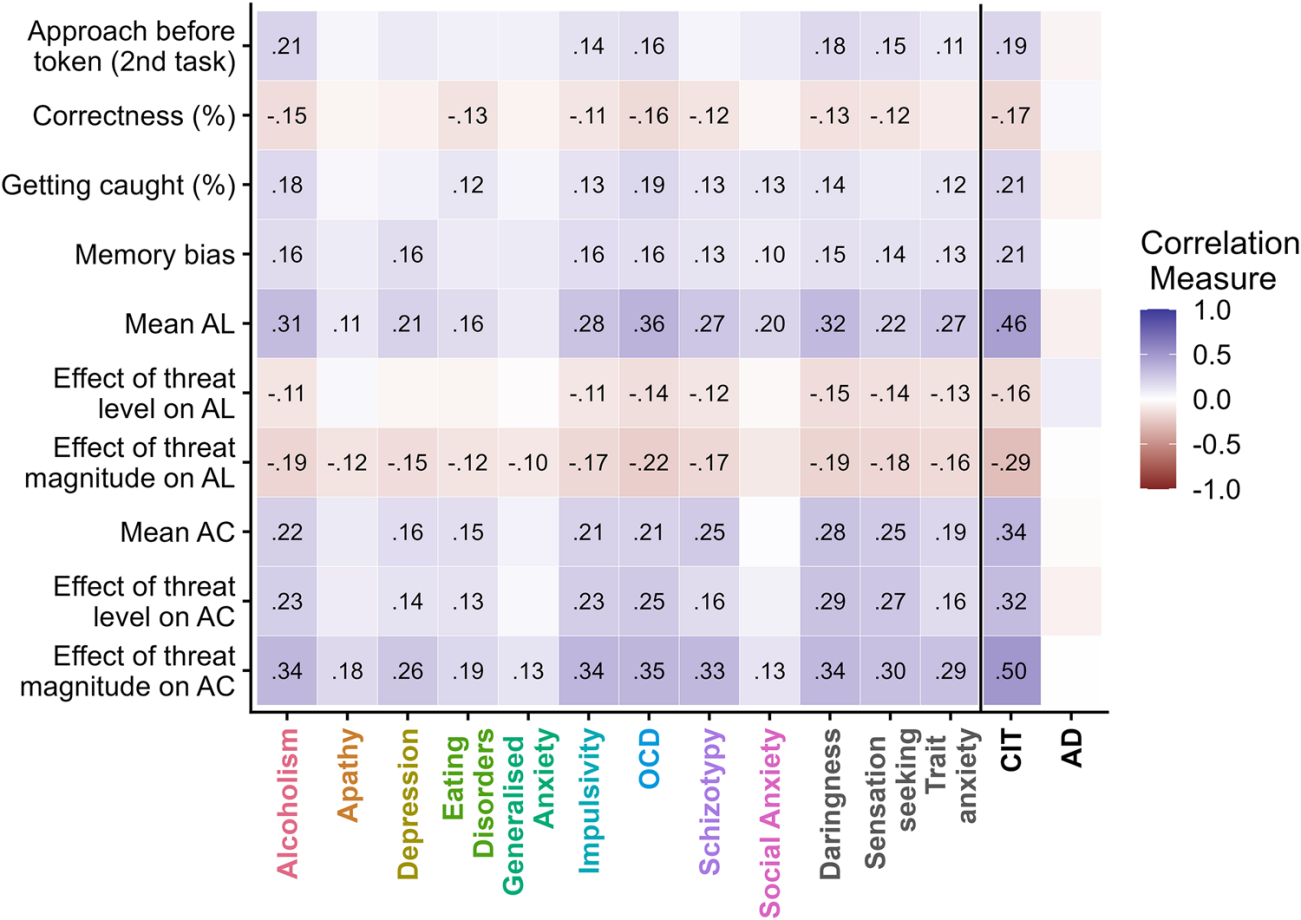
Correlation matrix between behavioural indices and questionnaires scores (left of black line) and symptom dimensions scores (right of black line). The colour scale and the numbers indicate the correlation coefficient. The number is only present when the absolute r > 0.10. This plot is based on the combined sample. AC Approach choices, AL approach latency.

Notably, some of the aforementioned relationships were profoundly non-specific, i.e. extended across multiple questionnaires. In a post-hoc analysis across the combined sample, 11/12 questionnaires had a positive relation at p < 0.05 with approach choices (see Table S3 and Fig. 4). Among those, sensation seeking and daringness had the strongest effect. In contrast, social anxiety had no effect. Similarly, 12/12 questionnaires had a significant positive relation at p < 0.05 with approach latency. Among these, OCD and daringness had the strongest effect. Importantly, the effect of CIT was larger than any of these individual questionnaires and accounted for the greatest proportion of variance. This exceeded the variance explained in daringness and OCD, the two questionnaires with the strongest relation to behaviour, at 21.2 and 21.1% respectively (see Table S3). Thus, while altered approach-avoidance behaviour broadly related to individual questionnaire scores, the strongest relation was not with anxiety questionnaires, and behaviour was better explained by a broad transdiagnostic dimension than by individual questionnaires.

### Subjective prior assumptions

**Fig. 5:**
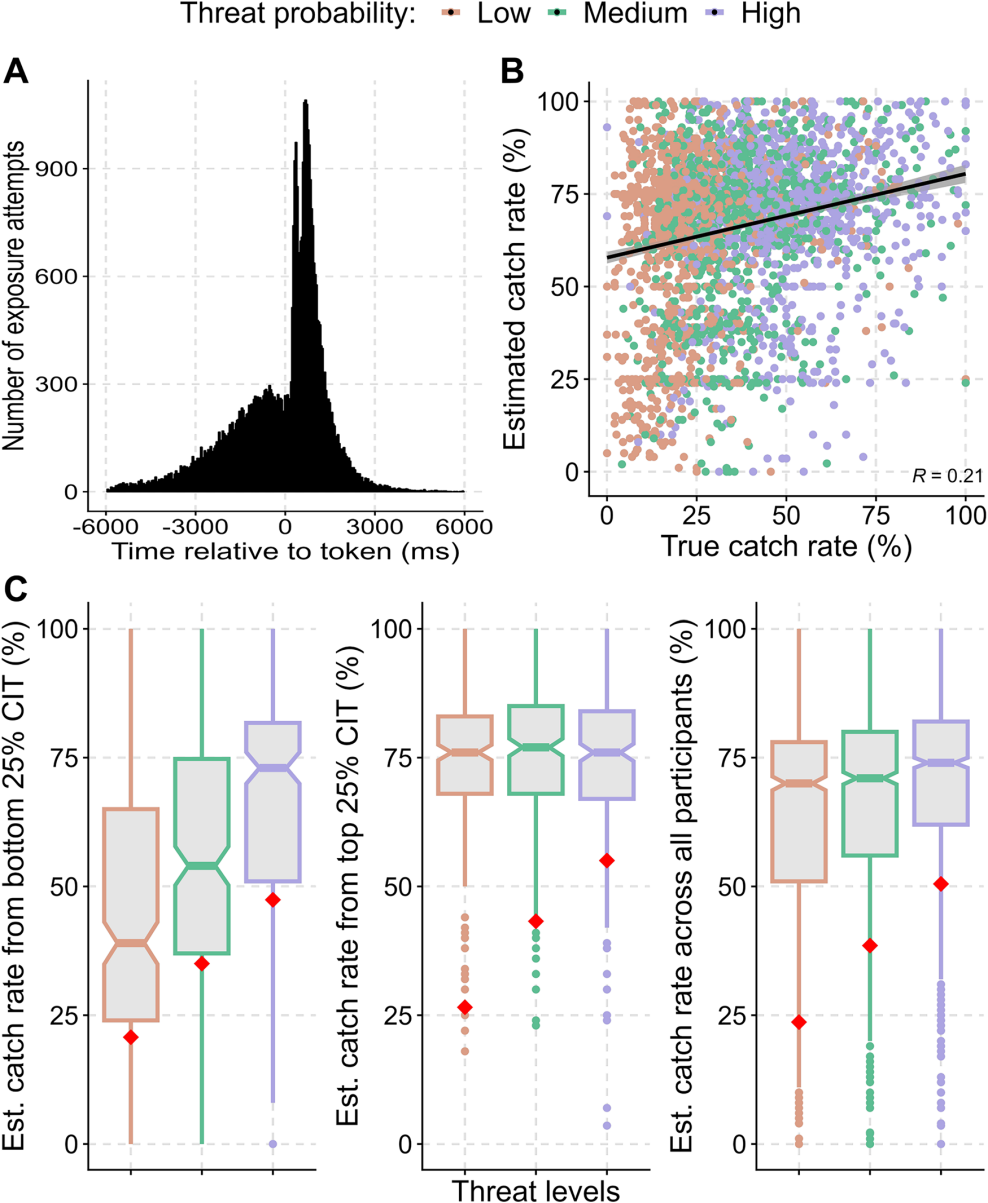
Subjective prior assumptions and threat memory. **A** Time of threat exposure attempts relative to token appearance. **B** Across participants, the estimated catch rates depended on true catch rate which had to be learned during the experiment. **C** CIT (Compulsive Behaviour and Intrusive Thought) is linked to biased threat memory such that the top 25% CIT scorers (centre) did not distinguish between different threat levels and overestimated their probabilities. While the bottom 25% CIT scores (left) and all participants (right) distinguished the threats better, they still overestimated the threat probabilities. Actual threat rates for each level are denoted by red diamonds. This plot is based on the combined sample, Figures S9 and S10 shows the results separately for the discovery and confirmation samples. Est.: estimated.

To investigate a subjective prior assumption that the presence of tokens alerts the predator, participants completed a predator exposure task. In the combined sample, the majority of the exposure attempts (58.8%) were made after the token had appeared, confirming previous work in lab-based settings (see Fig. 5A). People with higher CIT made more exposure attempts before the token appeared than those with low CIT (H18; p < 0.0001). A one standard deviation increase in CIT score corresponded to a 161.33 ms reduction in exposure latency. This suggests that people with high CIT might have a less strong prior assumption that the presence of rewards alerts the predator.

### Biased threat memory

After the behavioural tasks, participants estimated the probability of getting caught by each predator. As expected, the estimated catch rates depended on threat level (ANOVA: F(2, 1004) = 29.98, p < 0.0001) and true catch rates (LME: t(1, 2009) = 131.59, p < 0.0001) across all participants. However, the relation was far from perfect (regression coefficient β = 0.21), and there was a significant overall bias (t(1, 2009) = 4417.79, p < 0.0001): participants estimated catch rate on average 36.6% higher than the true catch rate (see Fig. 5B), which is in line with previous lab-based results [38].

CIT was linked to an even more biased threat memory: people with high CIT overestimated catch rates more, even while accounting for true catch rates (H16; p < 0.0001). Additionally, the estimated catch rates of people with high CIT depended less on actual catch rates (H17; p < 0.0001). Post-hoc exploratory analysis suggested that the effect of CIT was driven both by a stronger memory bias (β = 0.21, t(997) = 6.70, p < 0.0001) and lower memory precision (β = −0.08, t(997) = −2.54, p < 0.01) as indexed by the intercept and the slope relative to variations between estimated and actual catch rates. This indicates that persons with high CIT were significantly impaired in their ability to differentiate between threat levels. To illustrate this, the top 25% of CIT scorers did not distinguish the three threat levels (see Fig. 5C; see SI).

## Discussion

Behavioural cautiousness in AAC tasks is sensitive to anxiolytic drugs [2, 6] across species including humans [52, 53] but with no strong evidence of a relation to self-reported anxiety [2, 9, 19]. It also appears to be a stable behavioural trait [19, 23]. Here, we asked whether this behavioural predisposition is related to psychopathology dimensions in a cross-sectional and data-driven approach.

Our main result is that a broad transdiagnostic psychopathology factor (i.e. CIT) had the strongest relation to behavioural readouts. High CIT was related to decreased passive avoidance (i.e. lower avoidance rate), heightened behavioural inhibition (i.e. later approach), and decreased sensitivity to trial-by-trial threat characteristics such as probability and potential loss. Furthermore, we found that CIT was linked to a diminished belief that the presence of tokens alerts the predator, and a less precise representation of threat features, including overestimation of overall catch rates and less sensitivity to the catch rates of different threats.

Taken together, these results confirm previous findings that self-reported anxiety is not strongly related to behavioural cautiousness in AAC tests, and instead highlight the crucial impact of broad psychopathology, including daringness and compulsivity. Interestingly, this impact plays out on different levels and into different directions. First, despite overestimating threat probability, people with high CIT tend to approach more often. This is not adaptive, and indeed they get caught more often. Secondly, they tend to approach later. Such increased behavioural inhibition is traditionally thought to be an index of cautiousness in the same way as passive avoidance is [5], and as such increased approach and increased approach delay might appear to be in contradiction. However, there are alternative explanations for delayed approach. One would be a subjective belief in reward-threat correlations (Bach 2015); this however was reduced rather than increased in people with high CIT. A more likely explanation are longer decision times, due to less efficient decision strategies. This resonates with our third finding that people with high CIT are less sensitive to threat features, both in terms of their behaviour and in terms of explicit recollection of threat features, even though their higher approach rates meant they had more opportunity to learn the task statistics. Inefficient decision-making would be consistent with both findings and links with previous studies suggesting that individuals with compulsive disorders [54–56], and healthy people with high CIT [30] show reduced goal-directed control, and that compulsive behaviour relates to difficulty in building an accurate explicit model of the world [57] Furthermore, some studies hint towards the idea that model-based learning deficits in compulsive individuals predict the presence of habits [58]. In the context of behavioural inhibition, a similar mechanism might be at play at least to the extent that it seems to rely on a basic association between cues (i.e. rewards) and response (i.e. avoidance) that does not adapt to environmental characteristics (i.e. threat probability and magnitude). Furthermore, our findings revealed that higher IQ, generally associated with enhanced goal-directed control [59], was linked to higher integration of threat features into behaviour, and an increased belief in threat-reward correlations (see SI). These results, concordant with previous findings [19], suggest a form of attenuated behavioural inhibition, further endorsing the premise that variations in this behaviour could stem from differences in goal-directed control abilities. Furthermore, overestimation of threat has been implicated in the pathogenesis of OCD due to biased processing of threat-related information [60–63]. Our findings also relate to a recent study on risky foraging extended over time, in which some OCD symptoms showed associations with task measures [64].

As a limitation, our questionnaire battery did not include a systematic assessment of personality, such that we cannot dissect a relation to stable personality traits from potentially more dynamic psychopathology. Furthermore, our cross-sectional approach cannot unravel the causal and probably dynamic interplay of behavioural pre-dispositions, learning experiences, and their combined impact on psychopathology.

In sum, we add to a large body of literature suggesting that cautiousness in AAC tests, although these are often referred to as “anxiety tests”, does not specifically relate to self-reported anxiety [2, 9, 19]. While relations between some (albeit not all) anxiety-related questionnaires and AAC behaviour were found, these relations exhibited the same directional patterns as other psychiatric questionnaires, some of which consistently explained more variance than anxiety. Furthermore, our study found no connections with a transdiagnostic anxiety and depression dimension (AD), broadly characterised by apathy, depression, and general anxiety scores (see SI). These findings are unlikely to be due to the characteristics of the online sample, which was equally, if not more, anxious than in-person samples (see SI for details). In result, there does not appear to be a specific cross-sectional relation of behavioural cautiousness and anxiety [2, 19], and the relation of behavioural cautiousness on psychopathology might be more comprehensively understood through the lens of development, where behavioural cautiousness likely predisposes individuals to particular learning experiences. As a more proximal result, one might conclude that the specific AAC task used here, and potentially AAC tasks more broadly, offer limited clinical utility as behavioural diagnostics for anxiety disorders (although some AAC tasks have been suggested to predict treatment response [65]). Crucially, the present and several other tasks [2] are presented in a relatively abstract manner and thus are far removed from real-life settings. It is unclear to what extent the behavioural traits measured in such tasks relate to real-life behaviour. To advance our understanding, more ecologically valid tasks featuring realistic decision-making scenarios could bridge the gap between behavioral cautiousness observed in the laboratory and that displayed in everyday situations [66]. A complementary perspective is to focus on the exploration-exploitation dilemma inherent to real-life risky foraging settings but are largely absent from classical AAC implementations. Information seeking in such tasks might relate more closely to anxiety symptoms than behavioural cautiousness in AAC tasks [64, 67].

Beyond the specific findings of our study concerning behavioural inhibition and avoidance, our data underscore the advantages of employing transdiagnostic dimensions in contrast to traditional methods of phenotyping. Similar to previous research [57], we identified nonspecific patterns of correlation with task-related variables when we scrutinized commonly used yet infrequently compared clinical questionnaires. For instance, all twelve questionnaires consistently associated with behavioural inhibition, yet only the CIT factor distinctly aligned with this association and exhibited a larger effect than any individual questionnaire. Moreover, our findings affirm the generalisation of these symptom dimensions to another independent dataset, reinforcing their utility across various experimental designs [30, 31, 57, 68–71].

An important consideration is whether CIT might in fact represent a general factor of psychopathology (i.e. p-factor) [72, 73], given the broad and relatively unspecific loadings onto this factor. However, the strong correlations between our loadings and those identified in prior studies which delineated distinct cognitive effects, make this interpretation of CIT as a p-factor unlikely. Additionally, a second-order principal component, which might represent a broad-spectrum p-factor [73, 74], did not explain behaviour better than CIT, and several individual questionnaires (see SI). Yet, it is likely that CIT is more general than the initially chosen name suggests.

While web-based data collection offers many benefits in psychiatry research [75–77], concerns regarding data quality—particularly on mTurk—have been on the rise [34–37]. For example, our sample was characterized by scores above the threshold for further investigation on an alcohol use disorder screening instrument [78]. Also, our exclusion rate was somewhat higher than the typical range found in a meta-analysis, althouth not unusual for such studies [33]. Interestingly, Zorowitz et al. (2023) demonstrated that inattentive responding can lead to spurious correlations [37]. Our supporting analyses addressed potential sources of spurious correlations, making it unlikely that our results are due to inattentive responding (see SI for more details).

In conclusion, we find that a transdiagnostic symptom dimension has the strongest relation to cautious behaviour in an AAC task. Even if AAC tests have been extensively used to characterise the effects of anxiolytic agents and probe neural circuitry related to anxiety [2], they might not specifically relate to self-reported trait anxiety.

## Supplementary information

SI

## Data Availability

Curated CSV data and raw JSON data are available on OSF (https://osf.io/r8bdn/).

## Code availability

Code for task presentation (written in the open-source JsPsych JavaScript library) is available on OSF (https://osf.io/r8bdn/). All Matlab code used for pre-processing and R code used for analysis is publicly available on OSF (https://osf.io/r8bdn/).
