# Supplementary material for: Human approach-avoidance conflict behaviour relates to transdiagnostic psychiatric symptom dimensions
Source: Transl Psychiatry. 2026 Jan 28;16:61. doi: 10.1038/s41398-026-03835-8 (PMC12873436; doi:10.1038/s41398-026-03835-8)
Supplement: Supplementary file 1 — SI [file 41398_2026_3835_MOESM1_ESM.pdf]

Human approach-avoidance conflict behaviour relates to transdiagnostic psychiatric symptom dimensions

Juliana K. Sporrer, Filip Melinščak, Dominik R. Bach

Supplemental Methods

Attention checks

In an attempt to identify inattentive participants, we included several attention checks within the questionnaires. Namely, we asked:

- *"If you were paying attention to the previous questions, please select "A lot" as your answer." in OCI-R assessing* obsessive-compulsive disorder (Obsessive-Compulsive Inventory-Revised; Foa et al., 2002)
- *"It is important that you carefully read the following options, choose "Yes" below." in the SSMS assessing* schizotypy (Short Scales for Measuring Schizotypy; Mason et al., 2005).
- *"If you were focusing on the statements above, tick "A little"". in STICSA assessing* trait anxiety (State-Trait Inventory for Cognitive and Somatic Anxiety; Ree et al., 2008),

Each statement was formulated such that participants could not easily search for a recurrent keyword. Participants were told that if they missed the attention check questions, it would disqualify them from obtaining any potential bonuses that they may have won during the task.

Task details

For the AAC task, 144 trials were presented in randomised order, evenly distributed across 6 different levels of potential token loss, and 3 different threat probabilities. The token appeared on either side after an interval that was the sum of a fixed delay (500 ms) and a random sample from a truncated gamma distribution (k = 2, theta = 1, mean = 2 s, truncated at 6 s). The wake-up of the predator followed a Bernoulli process, independently determined in successive time bins of 20 ms. Using values established in previous work (Bach, 2015), the catch probabilities were $p_1 = .1$, $p_2 = .2$, or $p_3 = .3$ if the player was outside of the safe place for 100 ms. The actual catch rates depend on the participants' individual return latencies. In all cases, the trial ended 1 s after the pre-determined token disappearance time.

The second block comprised 57 trials from a different task, the predator exposure task, randomly interspersed with 15 AAC task refresher trials. As the graphical set-up is similar, the type of task was signalled with either a grey token on AAC task trials or a grey circle for predator exposure task trials.

Pre-processing

Data were pre-processed according to a pre-defined pipeline. To avoid response latencies being biased by lapses or extreme values, they were only included if they fell into response windows of 150 ms < approach latency < 2,000 ms and 0 ms < return latency < 2,000 ms, as in previous work (Bach, 2015, 2017), and were then log-transformed.

Factor Analysis

To replicate a previously established latent transdiagnostic structure (Gillan et al., 2016; Hopkins et al., 2022; Rouault et al., 2018), we applied a factor analysis with Maximum Likelihood Estimation using the *fa*() function from the *Psych* package in R with an oblique rotation (*oblimin*). We selected the number of factors based on Cattell's criterion (Cattell, 1966) using the Cattell-Nelson-Gorsuch (CNG) test from the *nFactors* package. The participants' factor scores were estimated using the Thurstone method. The CNG test revealed a 3-factor latent structure which was concordant with previous studies (Gillan et al., 2016; Hopkins et al., 2022; Rouault et al., 2018).

To ensure that our extracted latent structure replicated previous findings, we compared the item loadings and participants' z-scored scores using the item weights between our current study and that of Hopkins et al. (Hopkins et al., 2022) who had access to a substantially higher subject-to-variable ratio (N = 4782).

A difference between our sample and previous ones is the higher loading of alcoholism on the CIT. As addiction is known to strongly relate to compulsive behaviour, this deviation is unlikely to skew the interpretation of our results.

Adding three additional questionnaires to the factor analysis

The three additional questionnaires - daringness (CADS, Lahey et al., 2010), sensation seeking (BSSS, Hoyle et al., 2002), and trait anxiety (STICSA, Ree et al., 2008) - did not alter the 3-factor latent structure. In fact, we found close to perfect correlations between loadings and factor scores from the factor analysis including the same questionnaire set as in Rouault et al. (2018) and Gillian et al. (2016) and from the factor analysis including the additional questionnaires (Figure S3 – S6).

However, all three questionnaires loaded highly on the CIT factor and only marginally on AD and SW. While it was anticipated that sensation seeking and daringness would align closely with the CIT factor, we had predicted that trait anxiety would be more closely associated with the AD factor. Several factors might account for why trait anxiety showed greater relevance to CIT rather than AD. First, STICSA contains quite a few items that are linked to the intrusive nature of anxiety-related thoughts (e.g. item 10: "I can't get some thought out of my mind.", item 16: "I keep busy to avoid

uncomfortable thoughts.”, item 17: "I cannot concentrate without irrelevant thoughts intruding.” and item 19: “I worry that I cannot control my thoughts as well as I would like to.”. Second, the AD factor might relate more closely to apathy and depression rather than anxiety. Indeed, generalised anxiety as assessed by the STAI and which mapped more strongly onto AD than CIT, correlated more with apathy (discovery: r=.70, confirmation: r=.55) and depression (discovery: r=.79, confirmation: r=.72) than with STICSA scores (discovery: r=.62, confirmation: r=.68; see figure S3).

Second-order Principal Component Analysis

To test whether the effect of the CIT symptom dimension could be explained by a general psychopathology factor broader than CIT we tested the effect of a second-order factor. Second-order factor analysis can highlight higher-order, broad-spectrum factors by analysing associations between the oblique first-order factors (Caspi et al., 2014; Lahey et al., 2021).

To this end, we conducted a Principal Component Analysis (PCA) using the *prcomp*() function from the *Stats* package on the factor scores from the combined sample. We then tested the effect of the first component of the resulting PCA on behaviour.

While this second-order principal component significantly predicted behaviour, it did not explain behaviour better than the CIT symptom dimension. Indeed, behaviour only explained 13.49% variance in this second-order component, compared to 37.42% in CIT. In fact, it performed worse than 6 individual questionnaire scores, namely those assessing alcoholism, OCD, impulsivity, schizotypy, sensation seeking, and daringness (See table S3).

Supplemental Results

Higher anxiety levels in the online sample

The absence of anxiety's effect cannot be attributed to low anxiety levels within the online sample. Indeed, our current online participants exhibited higher anxiety scores compared to those in a previous in-lab study conducted by Sporrer et al. (2023). Specifically, the in-lab study reported mean trait anxiety scores (Ree et al., 2008) of 27.93 (SD = 5.57) for the discovery sample and 29.80 (8.89) for the exploratory sample. In comparison, the online sample presented considerably higher scores, with means of 39.38 (15.68) and 48.99 (14.66), respectively.

However, it is worth noting that the STICSA has a cut off at 43 to identify probable cases of clinical anxiety (van Dam et al., 2013), which is above the mean of the second sample. While it is possible that the second is more pathological it may also showcase underlying issue with data quality (see section below for more details).

Inattentive Responding and Data Validity

It is worth noting that the distribution of clinical scores differs significantly across waves in our study. Specifically, the confirmation sample exhibits more unusual patterns compared to the discovery

sample. For instance, scales assessing OCD and Generalized Anxiety lack the expected positive skew, and the Alcoholism scale displays bimodal distributions, both of which deviate from typical findings. Such atypical distributions have been linked to inattentive responding in questionnaires.

Zorowitz et al. (2023) demonstrated that inattentive responding can produce spurious associations between task behaviour and symptom measures. At first glance, one might think this could explain the lack of specificity in the pattern of cognitive deficits associated with CIT and other psychiatric questionnaires in our study. However, we believe several factors indicate this is not the case here.

First, Zorowitz et al. (2023) reported that nearly no significant (spurious) correlations emerged among symptom measures with more symmetric distributions. In our study, the strongest effects were observed in the discovery sample, where clinical scores followed typical patterns and were more symmetric (see Table 1, Figure S2). Second, Zorowitz et al. noted a marked reduction in significant correlations after excluding inattentive responders, a result unlikely to be due to reduced statistical power. In our experiments, we employed conservative, pre-registered exclusion criteria, combining several task-behaviour and self-report measures (see Methods). This approach, as noted by the authors, is one of the best ways to prevent spurious correlations. Although we used instructed items (e.g., "Please select ‘Strongly Agree’") rather than the recommended infrequency items (e.g., "I competed in the 1917 Summer Olympic Games"), we still believe our self-report screening was adequate. Additionally, rigorous participant screening does not appear to introduce overcontrol bias, as inattentive responding is independent of psychopathology (see Zorowitz et al., 2023).

Finally, Zorowitz et al. (2023) highlighted that false-positive rates for spurious behavioural-symptom correlations actually increase with sample size (i.e. keeping the inattentive responders) due to an increase in measurement bias rather than measurement noise. In our study, when all participants were included in the analysis, the results of interest were smaller or insignificant compared to those excluding inattentive respondents. Based on these additional tests and the successful replication of results, we can conclude that our findings are unlikely to be driven by false-positive correlations.

Interindividual differences in subjective prior assumptions

To investigate the subjective prior assumption that the presence of tokens alerts the predator, participants completed a predator exposure task. Here, optimal behaviour according to the task statistics was to make an exposure attempt early in the trial, regardless of token appearance. Interestingly, post hoc analysis on the combined sample show that the opposite pattern is true for people with high IQ who tried to tried to expose the robber more frequently after the token appeared (β = -2.1, $t(1, 961)$ = -2.06, $p<.05$). A one standard deviation increase in IQ score is linked to a 76.98 ms later approach. This suggests that people with high IQ might assume that the presence of tokens alerts the predator.

Interindividual differences in threat memory bias and precision

To further investigate which aspect of threat memory is influenced by factor scores, we extracted each participant’s slope and intercept from a linear regression between the estimated catch

rates and the threat levels, indicating threat memory precision and bias, respectively. We then used these regressors as dependent variables in another linear regression with the factor scores or demographics as predictors.

In this post-hoc exploratory analysis, CIT was associated with higher bias ($\beta = .21$, $t(997) = 6.70$, $p<.0001$) and lower precision ($\beta = -.08$, $t(997) = -2.54$, $p<.01$). The inverse was true with people with high IQ who had a lower bias ($\beta = -.12$, $t(1002) = -4.08$, $p<.001$) but a higher precision ($\beta = .09$, $t(1002) = 2.86$, $p<.005$).

We then split the data into subsets comprising of either participant scoring in the 25% top or the 75% bottom of CIT scores. We then repeated our analyses in these sub-samples. The 25% highest CIT scorers were not able to dissociate between the threats and reported similar catch rates ($F(2, 249) = 1.59$, $p>.05$) in contrast to the 75% lowest CIT scores who could ($F(2, 749) = 37.01$, $p<.0001$).

Table S1. Demographic and questionnaire scores within each sample. In parentheses are the standard deviation from the mean.

	Discovery sample	Confirmation sample
N	315	690
Female	149	338
Age	36.40 (11.01)	33.41 (9.89)
Generalized Anxiety	42.21 (10.80)	44.79 (8.36)
Eating Disorders	14.12 (9.83)	17.45 (9.92)
Apathy	35.39 (9.15)	38.20 (6.95)
Alcoholism	9.71 (9.35)	17.16 (9.41)
Depression	40.32 (10.04)	44.71 (8.13)
OCD	25.32 (16.45)	35.19 (13.73)
Social Anxiety	54.60 (31.53)	66.13 (28.27)
IQ	7.40 (3.55)	6.55 (2.91)
Impulsivity	62.12 (12.90)	69.3 (10.32)
Schizotypy	15.01 (9.20)	21.01 (8.51)
Sensation seeking	2.97 (0.84)	3.31 (0.72)
Trait anxiety	39.38 (15.68)	48.99 (14.66)
Daringness	2.55 (.79)	2.89 (.55)

Table S2. Behavioural results within each sample. In green are the data validation criteria that needed to be fulfilled to progress to the next analysis step. The GLMM and LMM included a 3 x 6 factorial design with threat level (low/medium/high) and potential loss (0-5 tokens). The models contained all possible polynomial terms, but we only reported linear contrasts for each factor or interaction. In parentheses are the standard deviation from the beta mean. The p-values are not corrected for multiple comparison and presented as a heuristic guide only.

	**Discovery sample**	**Confirmation sample**
Approach choices		
Threat level	$\beta = -.358$ (.02), $F(1, 44964) = 209.56$, $p<.0001$	$\beta = -.173$ (.02), $F(1, 98925) = 85.24$, $p<.0001$
Potential loss	$\beta = -2.329$ (.04), $F(1, 44964) = 3644.72$, $p<.0001$	$\beta = -1.003$ (.03), $F(1, 98925) = 1418.03$, $p<.0001$
Interaction: Threat level x Potential loss	$\beta = .052$ (.06), $F(1, 44964) = .69$, $p>.05$	$\beta = .018$ (.05), $F(1, 98925) = .16$, $p>.05$
Approach latency		
Threat level	$\beta = .010$ (.00), $F(1, 32333) = 11.8$, $p<.01$	$\beta = .003$ (.00), $F(1, 80454) = 3.89$, $p<.05$
Potential loss	$\beta = .043$ (.00), $F(1, 32333) = 104.41$, $p<.0001$	$\beta = .011$ (.00), $F(1, 80454) = 32.81$, $p<.0001$
Interaction: Threat level x Potential loss	$\beta = .002$ (.01), $F(1, 32333) = .08$, $p>.05$	$\beta = -.005$ (.00), $F(1, 80454) = 1.9$, $p>.05$
Withdrawal latency		
Threat level	$\beta = -.047$ (.01), $F(1, 22022) = 42.29$, $p<.0001$	$\beta = -.062$ (.00), $F(1, 51285) = 173.98$, $p<.0001$
Potential loss	$\beta = -.023$ (.01), $F(1, 22022) = 4.63$, $p<.05$	$\beta = -.012$ (.01), $F(1, 51285) = 3.06$, $p>.05$
Interaction: Threat level x Potential loss	$\beta = -.02$ (.02), $F(1, 22022) = 1.21$, $p>.05$	$\beta = .002$ (.01), $F(1, 51285) = .04$, $p>.05$

Table S3. Effect of each questionnaire scores (above black line) **and symptom dimension** (below black line) **on approach choices and latencies.** The last three questionnaire scores (i.e. sensation seeking, trait anxiety, and daringness) in grey were not included in the factorial analysis to calculate the symptom dimensions. This table is based on the combined sample. The p-values are not corrected for multiple comparison and presented as a heuristic guide only. See methods on how the explained variance was estimated.

**Questionnaire**	**Approach choices**	**Approach Latencies**	**Explained variance (%)**
Generalised Anxiety	β = 0.185 (0.09), $F(1, 44823) = 3.95$, $p<.05$	N.s.	2.13
Eating Disorders	β = 0.496 (0.14), $F(1, 44966) = 12$, $p<.001$	β = 0.044 (0.02), $F(1, 32335) = 3.98$, $p<.05$	5.57
Apathy	N.s.	N.s.	4.39
Alcoholism	β = 0.786 (0.11), $F(1, 44823) = 46.81$, $p<.0001$	β = 0.077 (0.02), $F(1, 32215) = 17.42$, $p<.0001$	17.46
Depression	β = 0.387 (0.09), $F(1, 44823) = 17.59$, $p<.0001$	N.s.	8.94
OCD	β = 0.708 (0.11), $F(1, 44823) = 44.88$, $p<.0001$	β = 0.09 (0.02), $F(1, 32215) = 28.63$, $p<.0001$	21.05
Social Anxiety	N.s.	N.s.	4.76
Impulsivity	β = 0.486 (0.1), $F(1, 44823) = 23.89$, $p<.0001$	β = 0.06 (0.02), $F(1, 32215) = 14.62$, $p<.001$	16.73
Schizotypy	β = 0.493 (0.1), $F(1, 44823) = 22.46$, $p<.0001$	β = 0.062 (0.02), $F(1, 32215) = 14.43$, $p<.001$	14.63
Sensation seeking	β = 0.65 (0.1), $F(1, 44823) = 43.96$, $p<.0001$	β = 0.081 (0.02), $F(1, 32215) = 26.96$, $p<.0001$	15.06
Trait anxiety	β = 0.662 (0.11), $F(1, 44823) = 35.38$, $p<.0001$	β = 0.053 (0.02), $F(1, 32215) = 8.81$, $p<.01$	12.64
Daringness	β = 0.602 (0.09), $F(1, 44966) = 48.42$, $p<.0001$	β = 0.096 (0.01), $F(1, 32335) = 50.34$, $p<.0001$	21.19
CIT	β = 0.666 (0.1), $F(1, 44521) = 48.54$, $p<.0001$	β = 0.078 (0.02), $F(1, 32000) = 25.86$, $p<.0001$	37.42

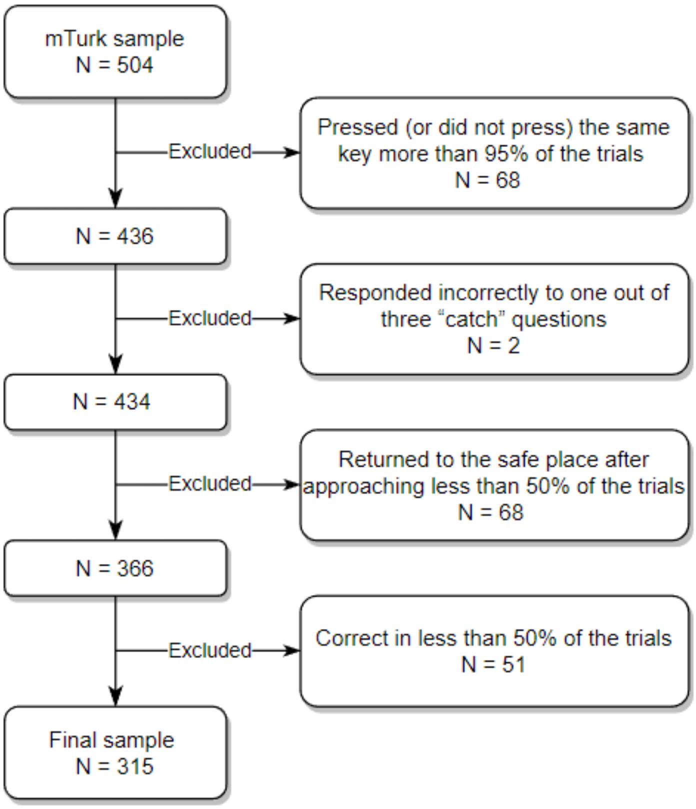

Figure S1. Exclusion flowchart for the first exploration experiment. The same criteria were applied to the second confirmation experiment.

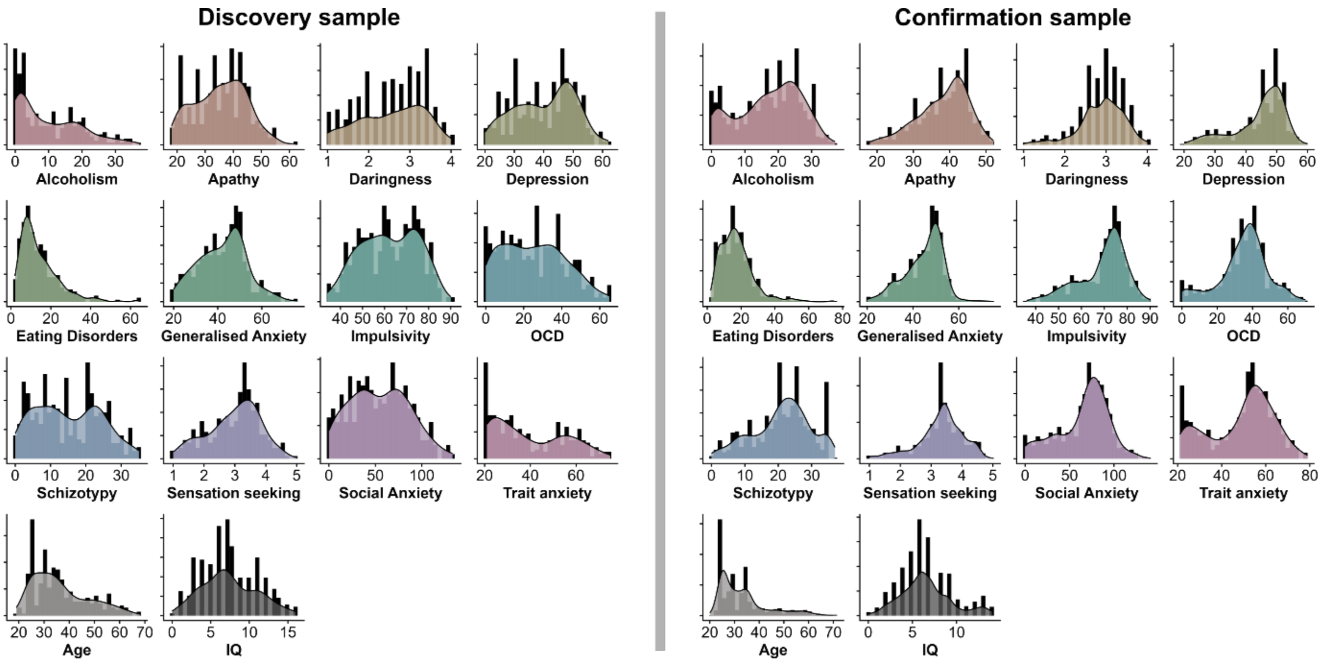

Figure S2. Distribution of questionnaire scores in both samples.

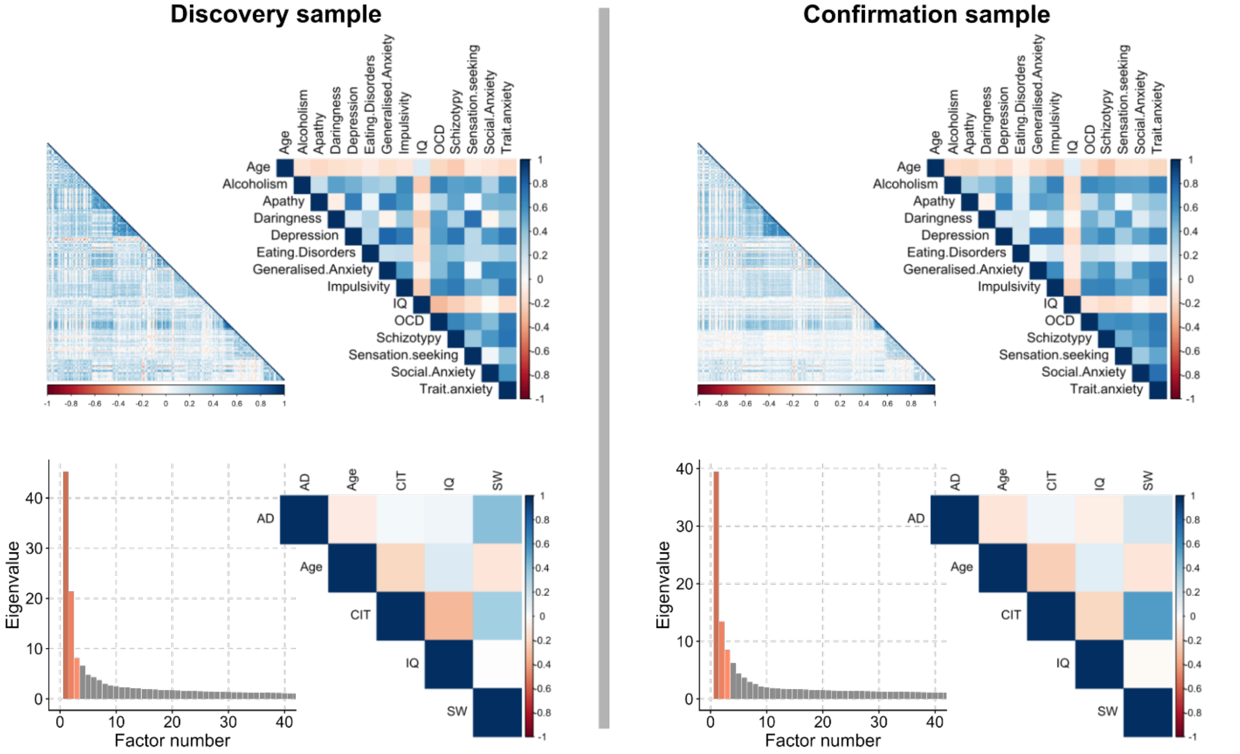

Figure S3. Correlation matrices between questionnaires in both samples. Specifically, the correlation between questionnaire items (top left), questionnaire scores and demographics (top right), and the three factors and demographics (bottom right). In the bottom left are the eigenvalues from the factor analysis revealing the three-factor solution that best accounted for our data. The colour scale indicates the correlation coefficient.

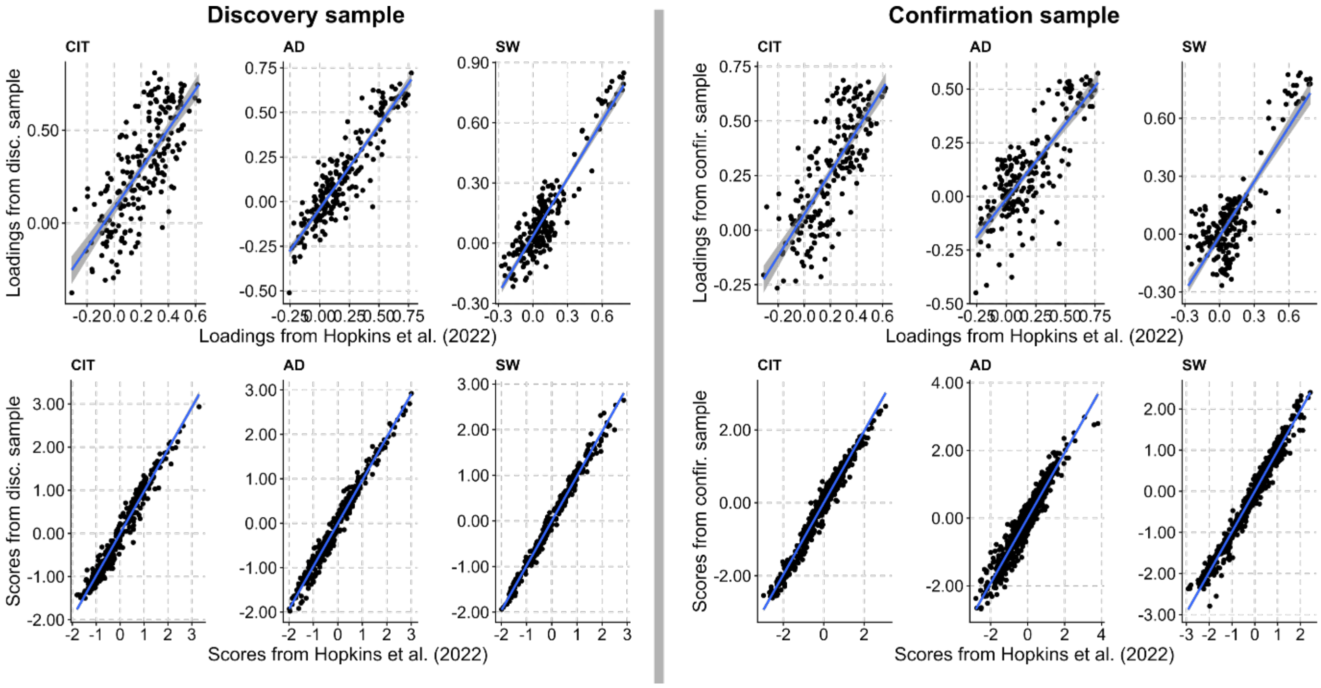

Figure S4. Correlations in both samples between current experiment samples loadings (top) **and scores** (bottom) **with Hopkins' et al. loadings and scores.** Hopkins et al. (2022) had access to a substantially higher subject-to-variable ratio (N = 4782).

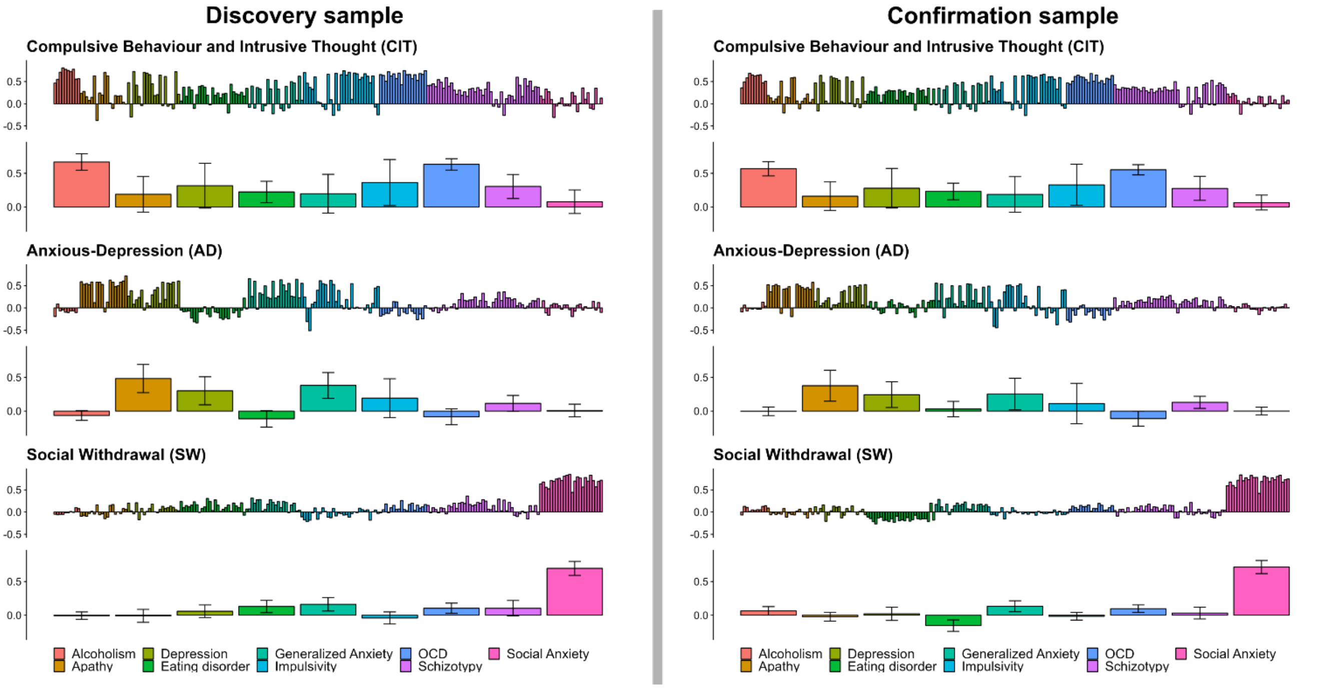

Figure S5. Questionnaire loadings onto each factor in both samples, color-coded by questionnaire. The top figures of each factor detail the loadings of the individual questionnaire items, the bottom figures summarise the loadings at the questionnaire score level. The error bars represent the standard deviation of the mean over item loadings.

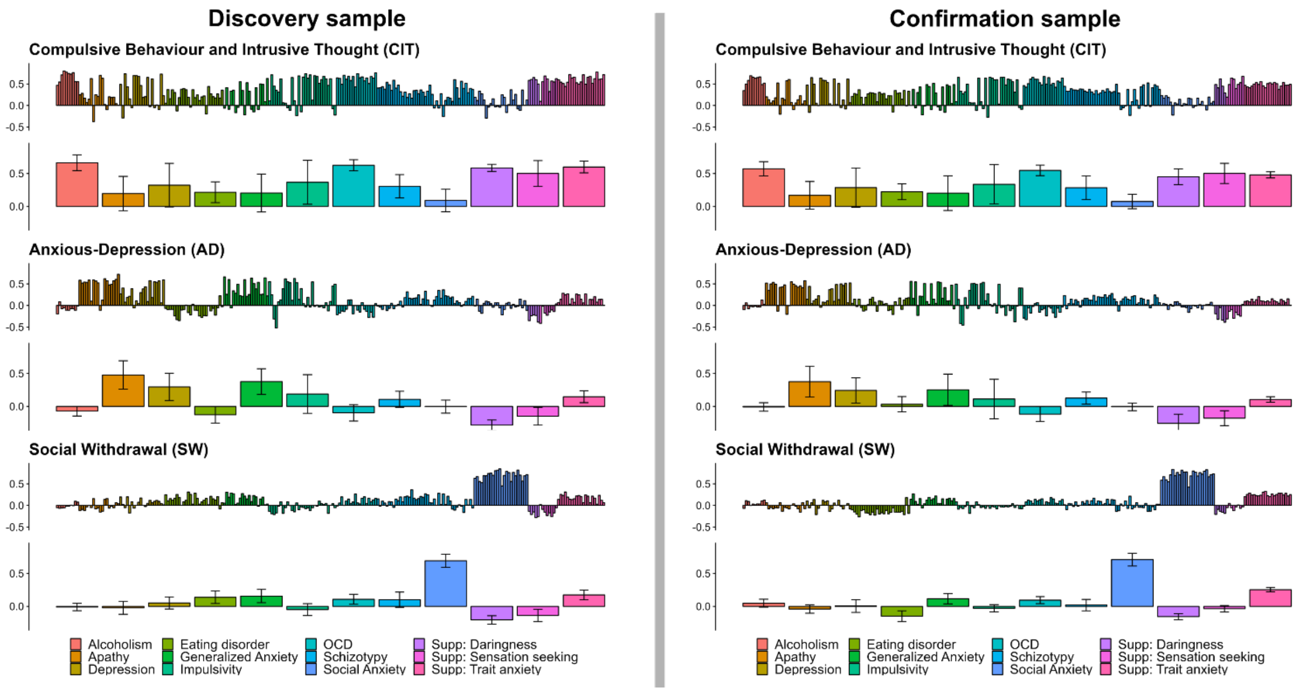

Figure S6. Loadings of all questionnaires (originals and additional) onto each factor in both samples, color-coded by questionnaire. The top figures of each factor detail the loadings of the individual questionnaire items, the bottom figures summarise the loadings at the questionnaire score level. The error bars represent the standard deviation of the mean over item loadings.

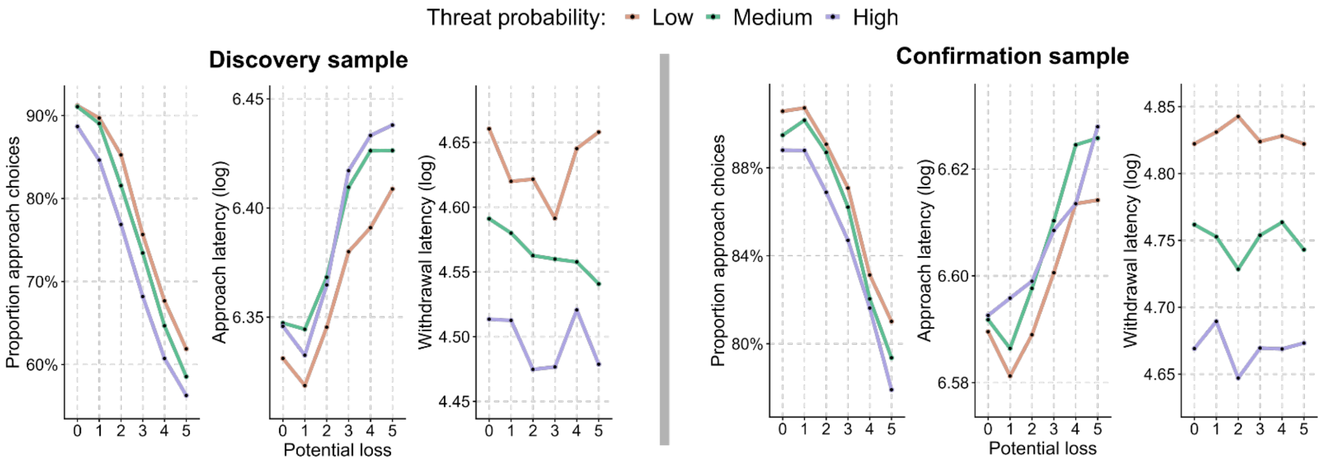

Figure S7. Behavioural results in both samples. Proportion of approach-avoidance decisions, indexing passive avoidance (left), approach latency, indexing behavioural inhibition (centre), and withdrawal latency (right).

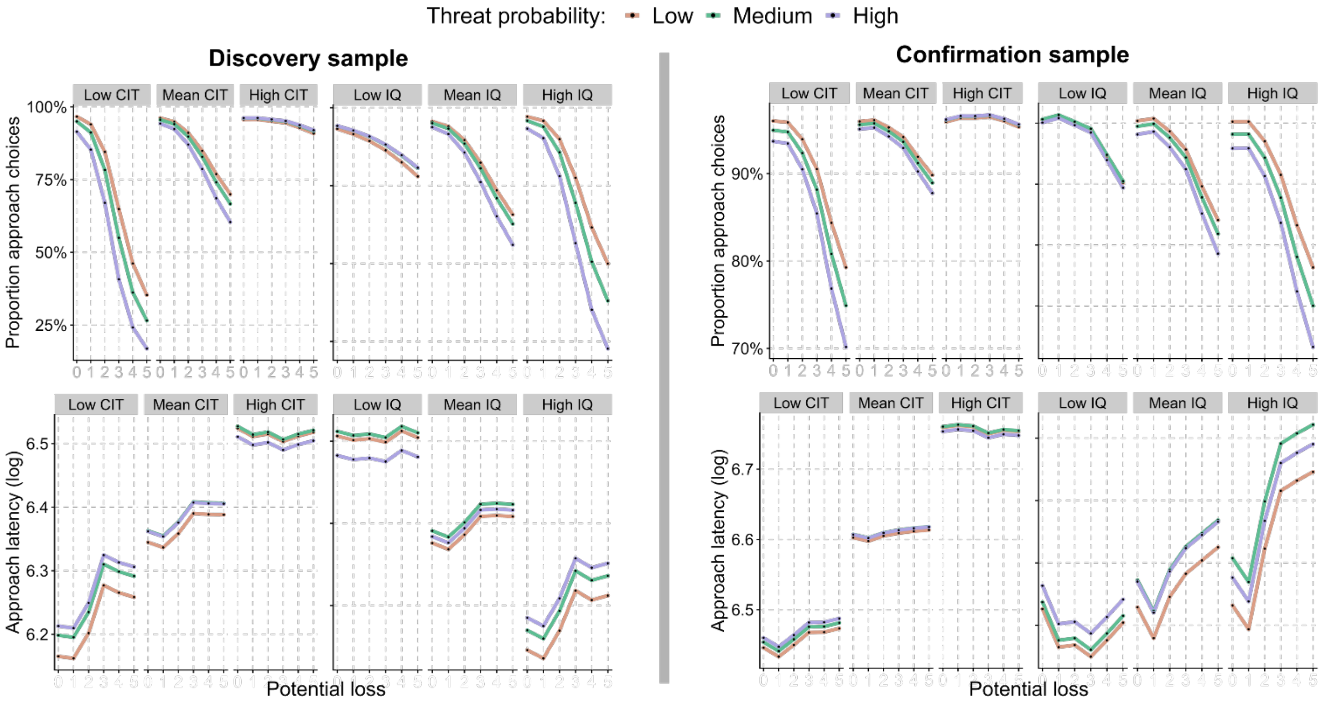

Figure S8. Estimated behavioural output according to main behavioural predictors in both samples. Estimated marginal means of approach choice (top) and latency (bottom) depending on CIT symptom dimension scores (left) or IQ (right) while other predictors are kept fixed. Low CIT/IQ: -1.5, Mean CIT/IQ: 0, and High CIT/IQ: +1.5

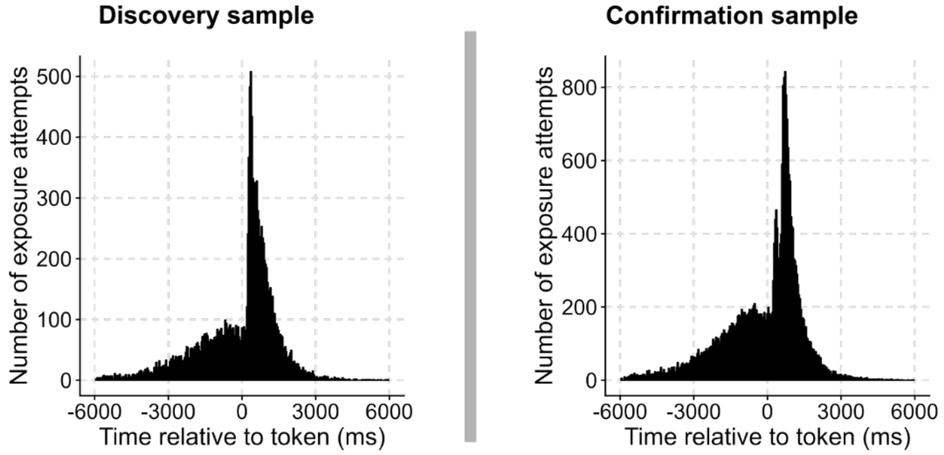

Figure S9. Time of threat exposure attempts relative to token appearance in both samples.

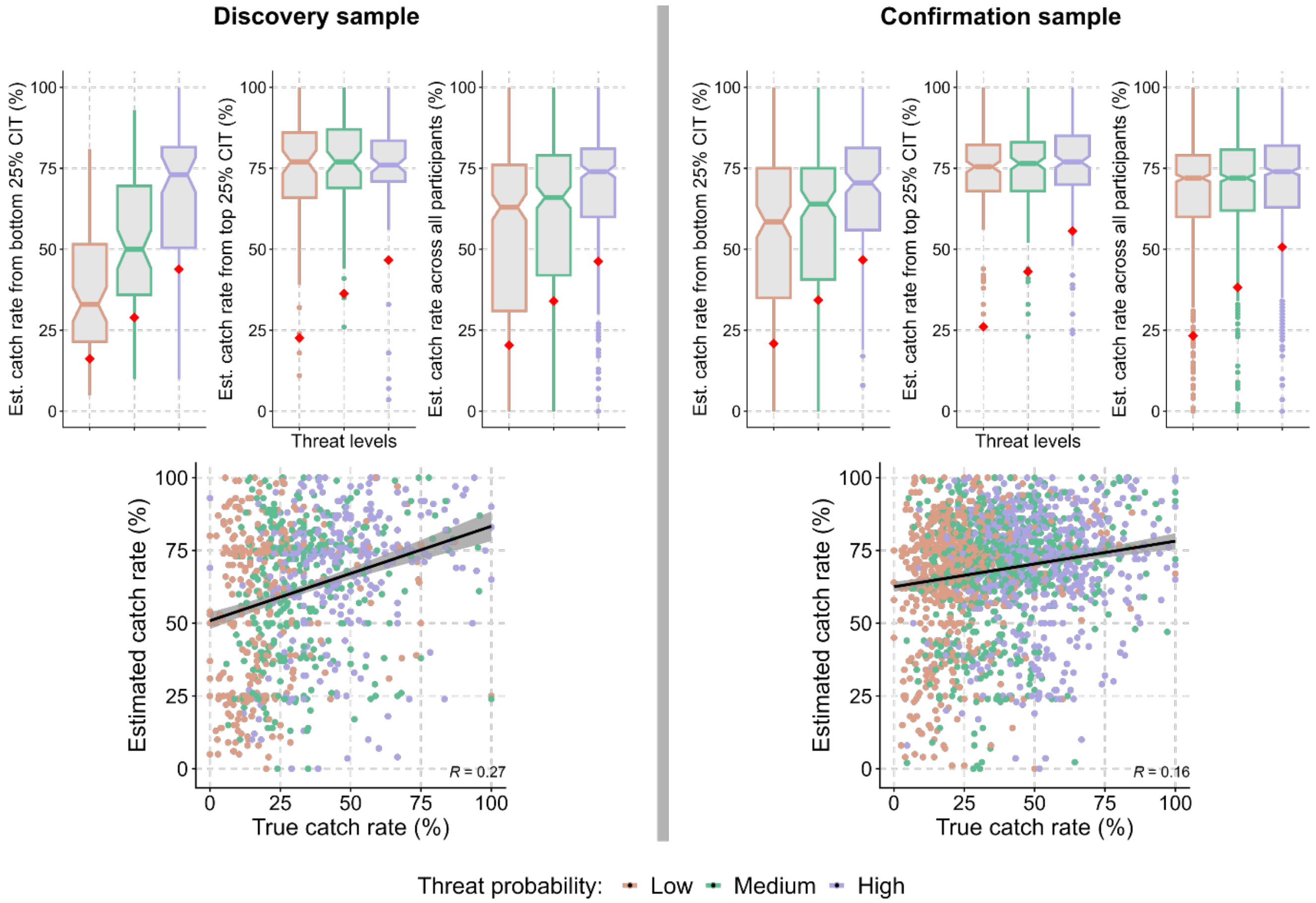

Figure S10. Biased reported threat memory in both samples. Across participants, the estimated catch rates depended on true catch rate which had to be learned during the experiment (bottom). CIT is linked to biased threat memory such that the top 25% CIT scorers (centre top) cannot distinguish between different threat levels and overestimate their probabilities. While the bottom 25% CIT scores (left top) and all participants (right top) distinguished the threats better, they still overestimated the threat probabilities. Actual threat rates for each level are denoted by red diamonds. Est.: estimated.

References

Bach, D. R. (2015). Anxiety-Like Behavioural Inhibition Is Normative under Environmental Threat-Reward Correlations. *PLoS Computational Biology*, *11*(12), 1–20. https://doi.org/10.1371/journal.pcbi.1004646

Bach, D. R. (2017). The cognitive architecture of anxiety-like behavioral inhibition. *Journal of Experimental Psychology: Human Perception and Performance*, *43*(1), 18–29. https://doi.org/10.1037/xhp0000282

Caspi, A., Houts, R. M., Belsky, D. W., Goldman-Mellor, S. J., Harrington, H., Israel, S., Meier, M. H., Ramrakha, S., Shalev, I., Poulton, R., & Moffitt, T. E. (2014). The p factor: One general psychopathology factor in the structure of psychiatric disorders? *Clinical Psychological Science*, *2*(2), 119–137. https://doi.org/10.1177/2167702613497473

Cattell, R. B. (1966). The Scree Test For The Number Of Factors. *Multivariate Behavioral Research*, *1*(2), 245–276. https://doi.org/10.1207/s15327906mbr0102_10

Gillan, C. M., Kosinski, M., Whelan, R., Phelps, E. A., & Daw, N. D. (2016). Characterizing a psychiatric symptom dimension related to deficits in goal-directed control. *ELife*, *5*. https://doi.org/10.7554/eLife.11305

Hopkins, A. K., Gillan, C., Roiser, J., Wise, T., & Sidarus, N. (2022). Optimising the measurement of anxious-depressive, compulsivity and intrusive thought and social withdrawal transdiagnostic symptom dimensions. *PsyArXiv*. https://doi.org/10.31234/osf.io/q83sh

Hoyle, R. H., Stephenson, M. T., Palmgreen, P., Lorch, E. P., & Donohew, R. L. (2002). Reliability and validity of a brief measure of sensation seeking. *Personality and Individual Differences*, *32*(3), 401–414. https://doi.org/10.1016/S0191-8869(01)00032-0

Lahey, B. B., Moore, T. M., Kaczkurkin, A. N., & Zald, D. H. (2021). Hierarchical models of psychopathology: empirical support, implications, and remaining issues. *World Psychiatry*, *20*(1), 57–63. https://doi.org/10.1002/wps.20824

Lahey, B. B., Rathouz, P. J., Applegate, B., Tackett, J. L., & Waldman, I. D. (2010). Psychometrics of a self-report version of the child and adolescent dispositions scale. *Journal of Clinical Child and Adolescent Psychology*, *39*(3), 351–361. https://doi.org/10.1080/15374411003691784

Ree, M. J., French, D., MacLeod, C., & Locke, V. (2008). Distinguishing Cognitive and Somatic Dimensions of State and Trait Anxiety: Development and Validation of the State-Trait Inventory for Cognitive and Somatic Anxiety (STICSA). *Behavioural and Cognitive Psychotherapy*, *36*(03), 313–332. https://doi.org/10.1017/S1352465808004232

Rouault, M., Seow, T., Gillan, C. M., & Fleming, S. M. (2018). Psychiatric Symptom Dimensions Are Associated With Dissociable Shifts in Metacognition but Not Task Performance. *Biological Psychiatry*, *84*(6), 443–451. https://doi.org/10.1016/j.biopsych.2017.12.017

Sporrer, J. K., Brookes, J., Hall, S., Zabbah, S., Serratos Hernandez, U. D., & Bach, D. R. (2023). Functional sophistication in human escape. *IScience*, *26*(11). https://doi.org/10.1016/j.isci.2023.108240

van Dam, N. T., Gros, D. F., Earleywine, M., & Antony, M. M. (2013). Establishing a trait anxiety threshold that signals likelihood of anxiety disorders. *Anxiety, Stress and Coping*, *26*(1), 70–86. https://doi.org/10.1080/10615806.2011.631525

Zorowitz, S., Solis, J., Niv, Y., & Bennett, D. (2023). Inattentive responding can induce spurious associations between task behaviour and symptom measures. *Nature Human Behaviour*, *7*(10), 1667–1681. https://doi.org/10.1038/s41562-023-01640-7
